# Novel Phenotypes and Cardiac Involvement Associated With DNA2 Genetic Variants

**DOI:** 10.3389/fneur.2019.01049

**Published:** 2019-10-04

**Authors:** Ariadna González-del Angel, Michela Bisciglia, Steven Vargas-Cañas, Francisca Fernandez-Valverde, Ekaterina Kazakova, Rosa Elena Escobar, Norma B. Romero, Claude Jardel, Benoit Rucheton, Tanya Stojkovic, Edoardo Malfatti

**Affiliations:** ^1^Laboratorio de Biología Molecular, Departamento de Genética Humana, Instituto Nacional de Pediatría, Mexico City, Mexico; ^2^AP-HP, GHU La Pitié-Salpêtrière, Institut de Myologie, Paris, France; ^3^Instituto Nacional de Neurologia y Neurochirurgia, Mexico City, Mexico; ^4^Laboratorio de Patología Experimental, Instituto Nacional de Neurología y Neurocirugía, Mexico City, Mexico; ^5^Cedimemm: Centro de Diagnóstico en Metabolismo Energético y Medicina Mitocondrial, Mexico City, Mexico; ^6^Unit of Muscle Dystrophies, Instituto Nacional de Rehabilitacion (INR), Mexico City, Mexico; ^7^Sorbonne Université, INSERM, Centre de Recherches, Centre de Référence des Maladies Neuromusculaires Nord/Est/Ile de France, GHU Pitié-Salpêtrière, Paris, France; ^8^AP-HP, GHU La Pitié-Salpêtrière, U.F. Cardiogénétique et Myogénétique, Service de Biochimie Métabolique, Paris, France; ^9^Service Neurologie Médicale, Centre de Référence Maladies Neuromusculaire Paris-Nord, CHU Raymond-Poincaré, Garches, France; ^10^U1179 UVSQ-INSERM Handicap Neuromusculaire: Physiologie, Biothérapie et Pharmacologie Appliquées, UFR des Sciences de la santé Simone Veil, Université Versailles-Saint-Quentin-en-Yvelines, France

**Keywords:** mitochondrial disease, early onset myopathy, *DNA2*, cardiomyopathy, velopharyngeal weakness, rhabdomyolysis

## Abstract

**Objectives:** To report two novel *DNA2* gene mutations causing early onset myopathy with cardiac involvement and late onset mitochondriopathy with rhabdomyolysis.

**Methods:** We performed detailed clinical, muscle histopathology and molecular studies including mitochondrial gene NGS analysis in two patients (Patient 1 and 2), a mother and her son, belonging to a Mexican family, and a third sporadic French patient.

**Results:** Patient 1 and 2 presented with an early onset myopathy associated with ptosis, velopharyngeal weakness, and cardiac involvement. Patient 3 presented rhabdomyolysis unmasking a mitochondrial disease characterized by a sensorineural hearing loss, ptosis, and lipomas. Muscle biopsies performed in all patients showed variable mitochondrial alterations. Patient 3 had multiple mtDNA deletion in his muscle. Genetic studies revealed a novel heterozygous frameshift mutation in *DNA2* gene (c.2346delT p.Phe782Leufs^*^3) in P1 and P2, and a novel heterozygous missense mutation in *DNA2* gene (c.578T>C p.Leu193Ser) in the P3.

**Conclusions:** To date only few AD cases presenting either missense or truncating *DNA2* variants have been reported. None of them presented with a cardiac involvement or rhabdomyolysis. Here we enlarge the genetic and phenotypic spectrum of *DNA2*-related mitochondrial disorders.

## Background

DNA replication helicase/nuclease 2 (*DNA2*) encodes for a helicase/nuclease family member involved in the processes of mtDNA replication and the long-patch base-excision repair (LP-BER) (1). The protein contains a nuclease, and an ATPase and helicase domain. In mitochondria, DNA2 interacts with polymerase γ by enhancing its enzymatic activity (2). Furthermore, DNA2 co-localizes in nucleoids with Twinkle helicase (3), and its recruitment increases in mitochondria showing *PEO1* gene mutations, suggesting its essential participation in mtDNA maintenance and replication (4). Mutations in *DNA2* have been associated with autosomal dominant (AD) progressive external ophtalmoplegia (PEO) with multiple mtDNA deletions (MIM615156), and congenital onset myopathy with ptosis (5). Autosomal recessive (AR) *DNA2* mutations have been reported in Seckel syndrome type 8 (OMIM 615807), a condition characterized by intra-uterine growth retardation, dwarfism, mental retardation, microcephaly and facial dysmorphisms (6). Here we report three patients showing two novel phenotypes. Patient 1 (P1) and 2 (P2), presented with an early onset myopathy, velopharyngeal weakness, and cardiac arrhythmias. Patient 3 (P3) presented with a unique episode of rhabdomyolysis and had sensorineural hearing loss, ptosis, and lipomas.

## Case Presentation

**Figure 1:**
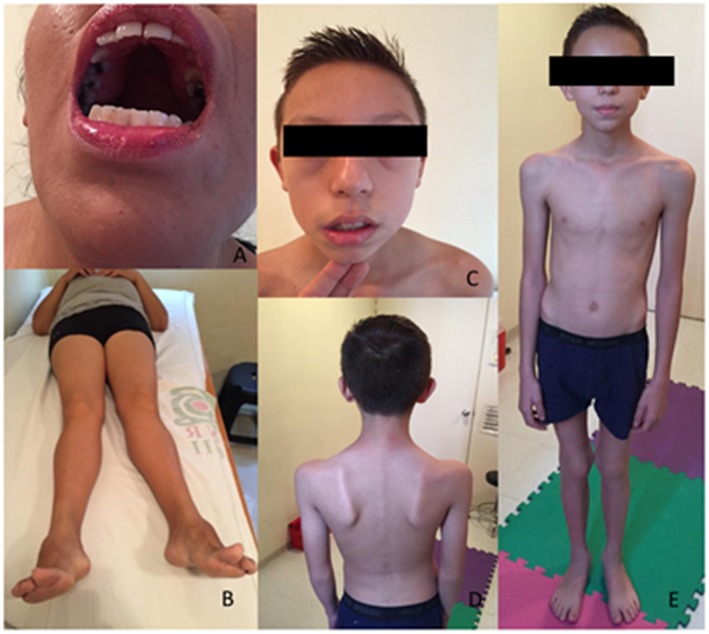
Patients features. P1, exanimated at the age of 44 years, presented a high arched palate **(A)**, a genu valgum and bilateral pes cavus **(B)**. P2, at the age of 11, showed a lower face weakness with a semi-open mouth **(C)**. He presented with scapular winging, a periscapular amyotrophy **(D)** and a thin muscle bulk **(E)**.

P1 is a female born to healthy non-consanguineous Mexican parents. Pregnancy was reportedly normal. She was born with breech presentation and presented neonatal hypotonia, failure to thrive and delayed motor milestones with gait acquisition at 3 years. She had frequent falls and reported episodes of diffuse myalgias during childhood. Running was impossible. She had poorly comprehensible speech, necessitating a retropharyngeal implant at 7 years. She also had difficulties in gaining weight with repeated episodes of transient weight loss. A right-sided scoliosis was discovered at 12 years and treated with a corset and physical therapy. Progressive difficulties in walking were reported since the age of 15 years. At 28 years an atrioventricular block, Mobitz type 2, was diagnosed and a pacemaker was implanted at the age of 35 years. She experienced a cardioembolic left parietal stroke during the pacemaker implantation leading to aphasia and dysgraphia. Clinical examination at age of 44 years showed facial asymmetry with prominent zygomatic right bone and facial weakness, high-arched palate (Figure 1A), and nasal voice. There was no ptosis or ophthalmoparesis. Manual muscular testing revealed diffuse upper and lower limbs proximal (4/5 MRC, Medical Research Council), and abdominal muscles (4/5 MRC). She had waddling gait and a Gowers' sign. There was no distal or axial weakness. She presented shoulder muscle wasting, right-sided kyphoscoliosis, *genus valgus*, and bilateral *pes cavus* (Figure 1B). Follicular hyperkeratosis was also noted. Deep tendon reflexes were absent and sensory testing was normal. She had no cataract, retinitis, or hearing impairment. Laboratory investigations showed normal CK level. Her cardiologic treatment includes amlodipine, metoprolol, and hydrochlorothiazide.

P2 is the son of P1 and was born at 38 weeks by caesarian delivery. Pregnancy was complicated by preeclampsia and a gestational diabetes. At delivery, he quoted 8 at Apgar score and was hypotonic, but he did not need intensive care unit or external ventilation. He successively showed failure to thrive and had delayed motor skills with gait acquisition at 18 months. During childhood he presented frequent falls especially when running. Mental confusion and weight loss during fever or viral infections were reported. Clinical examination at 11 years of age showed facial weakness with a semi-open mouth (Figure 1C), nasal speech with high-pitched voice, high-arched palate, and a mild ptosis. Extraocular movements were normal. Manual muscular testing revealed diffuse weakness quoting 3/5 MRC. Walking on toes was impossible. He presented Gowers' sign, scapular winging, pectoral, and periscapular amyotrophy were also noted (Figures 1D,E). CK level was slightly elevated at 334 U/I (21–232 U/I the normal range). His EMG and nerve conduction studies performed at 4 years disclosed a myopathic pattern. Somatosensory evoked potential and magnetic resonance of the brain, performed at 3 years, were normal. Electrocardiogram and echocardiogram were normal, but a Holter ECG revealed supraventricular tachycardia, treated with metoprolol.

The third patient (P3) is a Caucasian, 68 years old male with a long history of ischemic cardiopathy, treated by coronary stenting. His family history was irrelevant for neuromuscular disorders. His cardiologic treatment included atenolol, angiotensin inhibitor, and aspirin. He was under rosuvastatin by the age of 63 and was treated with bronchodilators and steroids for a chronic obstructive pulmonary disease. He presented a sensorineural bilateral hearing loss, discovered at the age of 55 years. Previous CK levels were normal. The patient had no history of muscular weakness until the age of 67 years, when he presented an episode of rhabdomyolysis (91 000 UI/l) following abdominal surgery for umbilical hernia and a concomitant transient episode of proximal limb weakness. Clinical examination 6 months later revealed bilateral ptosis, with no limitations in extraocular movements and normal muscle strength. The patient presented neck lipomas. His EMG and nerve conduction studies were normal. Six months later the CK level was normal.

## Laboratory Investigations and Diagnostic Tests

### Biopsy Findings

**Figure 2:**
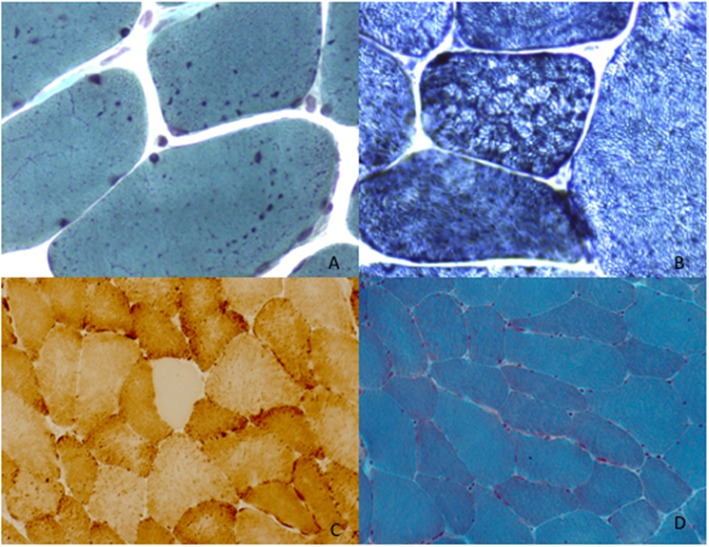
Muscle morphological studies. P1 left deltoid muscle biopsy at 34 years showed the presence of enlarged mitochondria with uneven distribution evident with Gomori trichrome staining (mGT) **(A)**. Oxidative reactions (NADH) demonstrated the presence of an altered mitochondrial network giving rise to lobulation and type 1 predominance **(B)**. P3 left deltoid biopsy at the age of 68 years old, revealed COX oxidative reaction **(C)** and the presence of mitochondrial aggregates evident with Gomori trichrome staining (mGT) **(D)**.

P1 underwent left deltoid muscle biopsy at 34 years of age showing mitochondrial aggregates evidenced by Gomori trichrome staining (mGT) (Figure 2A) and NADH oxidative reaction (Figure 2B). There was type 1 fibers predominance. Ultrastructural studies confirmed the presence of enlarged mitochondria with disrupted cristae without any alteration of the myofibrillar structure. P2 underwent left deltoid muscle biopsy at 11 years of age. The biopsy showed mild fiber size variability and normal nuclear positioning. Oxidative staining revealed the presence of type 1 fibers uniformity and rare areas of uneven oxidative reactions (Figure 2B). Ultrastructural studies showed mitochondria with altered cristae. P3 underwent left deltoid biopsy at 68 years revealing loss of COX activity in some fibers (Figure 2C), a lobulated aspect of several muscle fibers and the presence of subsarcolemmal mitochondrial aggregates (Figure 2D).

### Genetic Analysis

Peripheral blood leukocyte genomic DNA from the patients was analyzed by a next generation sequencing panel (NGS), Roche NibelGen, Madison, WI, USA, comprising the entire coding region and exon–intron junctions of 244 genes associated with mitochondrial diseases. The complete list of all genes is reported in Table A1. More than 95% of the nucleotides within the exons in the targeted regions were interrogated at more than 30 × depth of coverage. Variants were annotated and evaluated by cross-referencing to the Single Nucleotide Polymorphism Database (dbSNP), Human Genome Variation Society (HGVS) nomenclature. Mutation pathogenicity was predicted through 3 *in silico* programs: SIFT, AlignGVGD, Polyphen2, and Mutation Tester. The sequence variants were interpreted and classified based on recommendations from the French Association of Molecular Genetics Practitioners. The list of VUS found all patients is reported in Table A2.

NGS panel performed on P1 and P2, revealed a novel, heterozygous variant, c.2346delT; p.Phe782Leufs^*^3, in exon 15 of *DNA2* gene. The variant results in a change from phenylalanine to leucine at amino acid position 782, and a shift in the reading frame thereafter. The Phe782 residue is located in the ATPase and Helicase domain and it is conserved in both mammalian and yeast DNA2. A termination codon is predicted 3 codons beyond this change. This variant has not been reported in the literature and is not present in the population database dbSNP. The possible consequence of the production of premature termination codons is the degradation of mutant mRNA by nonsense-mediated decay and haploinsufficiency with reduced DNA2 synthesis.

P3 harbored a novel, c.578T>C p.Leu193Ser, heterozygous missense mutation in *DNA2* gene. Although this variant is considered to be of uncertain pathogenicity according the ACMG criteria, several elements suggest a damaging effect. First of all, the variant results in a change from leucine to serine at amino acid position 193, the residue is highly conserved in both mammalians and yeast. Furthermore, the physico-chemical differences between these two amino acids are very important as Grantham's distance is 145 and the change is a switch between a hydrophobic aliphatic amino acid by a hydrophilic amino acid. This variation is in the helicase domain and is predicted to be pathogenic through AlignGVGD, and MutationTester *in silico* programs. Additionally, it is predicted to be probably damaging with a score of 0.998 by Polyphen2.

**Figure 3:**
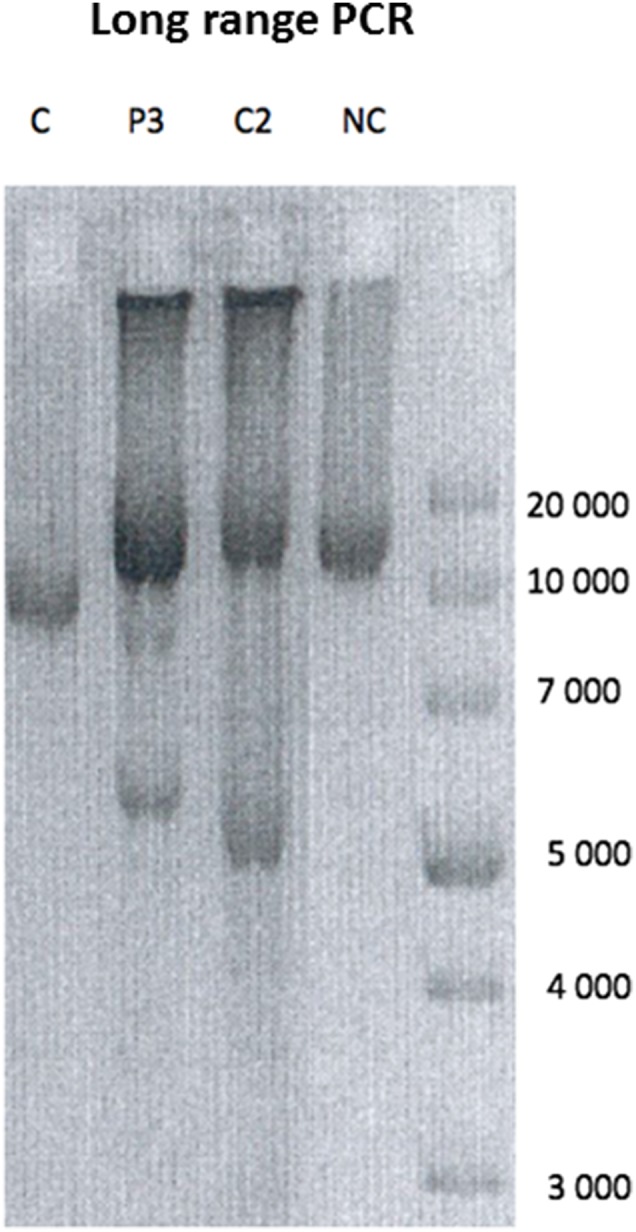
Long range PCR. Long range PCR showing multiple deletions on muscle mtDNA in P3 compared to a patient showing a single mtDNA single deletion (C), a *POLG* mutated patient with a similar age showing multiple deletions (C2), and a normal control (NC).

Both the variants have not been reported before. P1 and P2 variant is classified as pathogenic due to very strong criteria according to the French Association of Molecular Genetics Practitioners. P3 is considered possibly pathogenic. The variants have been submitted to Clinvar in keeping with the recommendation of the Human Variome Project (http://www.ncbi.nlm.nih.gov/clinvar/). It was not possible to test the presence of multiple deletion on skeletal muscle or urinary epithelial cells. In contrast we demonstrated the presence mtDNA multiple deletions on DNA extracted from skeletal muscle in P3 (Figure 3). The abnormalities observed in our patient were absent in muscle DNA from a 45 years healthy control suggesting that they are pathologic (Figure 3). As an age-matched muscle DNA was not available we used a DNA sample extracted from muscle of a 45 years subject as a normal control (Figure 3). Furthermore they were similar in intensity to those observed in the multiple deletions control (patient with *POLG* known mutations aged of P3) (Figure 3).

## Discussion

*DNA2* gene associated mitochondrial myopathy was firstly described in four patients by Ronchi et al. (7) as an adult onset skeletal myopathy involving the limb-girdle and lower limbs, with ptosis and ophtalmoplegia and *DNA2* missense mutations. A congenital presentation was successively reported in association with a truncating mutation (5, 8) suggesting that truncating mutations resulting in haploinsufficiency of DNA2 might lead to more severe phenotype with congenital presentation (5). In contrast, the residual function of helicase and ATPase activities due to missense mutations may account for the late onset cases.

Here we report two novel mutations in *DNA2* gene. Although these variants fit with the clinical and histopathologic features of patients, we cannot completely exclude that additional variants of interest may have been detected with more comprehensive sequencing (e.g., exome), given the massive heterogeneity of genetic muscle diseases. P1 and P2 presented the c.2346delT, p.Phe782Leufs^*^3 frameshift mutation localized in the distal part of DNA2 ATPase and helicase domain causing an early onset myopathy with ptosis, velopharyngeal weakness and cardiac disease of the conduction system and rhythm. This variant, although functional test were not performed, could cause an enhanced helicase activity as *DNA2* demonstrated by Ronchi et al. in a patient with adult onset myopathy harboring a p.Val723Ile variant. Since helicase and nuclease activity seems to be coupled (9), uneven helicase activity might have a deleterious effect on affected cells. This enhanced helicase activity could be responsible for the presence of enlarged mitochondria in the muscle biopsy of P1. Furthermore, impaired helicase activity alone seems to be more prone to produce double-strand breaks than mutant helicase and nuclease activities together (10). This unbalanced enzymatic activity could be an explanation for the fact that we failed to find any mtDNA deletion in blood samples both from P1 and P2. Unfortunately, we did not have access to spare skeletal muscle to search for mtDNA deletions.

When considering the cardiac involvement, P1 presented a second-degree atrioventricular block Mobitz type 2 leading to a pacemaker implantation and P2 had a supraventricular tachycardia medically treated. Incidence of cardiac complications is frequent in patients with mitochondriopathy, either in isolation, or as a component of a multisystem disease (7, 11). Cardiac involvement in mitochondriopathies is heterogeneous, ranging from conduction system disease and rhythm disease, to myocardial abnormalities such hypertrophic and dilated or non-compaction cardiomyopathies (12, 13). Cardiac involvement in nuclear genes implicated in mtDNA maintenance and repair includes sinus bradycardia, ischemic heart disease, atrial arrhythmias, conduction defects, and dilated cardiomyopathy, such has been described in Twinkle-related PEO1 mutations (14). Pathogenic *OPA1* mutations have been associated with fatal hypertrophic cardiomyopathy (15). From a clinical point of view P1 and P2 presented a clinical phenotype compatible with a congenital myopathy with cardiac involvement. However, the muscle pathologic findings pointed specifically toward a mitochondrial disorder and detailed ultrastuctural analysis with electron microscopy failed to show any structural abnormality reminding of a congenital myopathy.

Patient 3 showed a milder phenotype and a late onset mitochondrial disease, associated with the c.578T>C p.Leu193Ser leading to the development of mtDNA multiple deletions in muscle (Figure 3). This variant, although functional tests were not performed, seems to behave as the variants described by Ronchi et al. (7) causing adult onset limb-girdle myopathy with ptosis and ophtalmoplegia and mtDNA multiple deletions. As far as we know, rhabdomyolysis is a quite exceptional complication in mitochondriopathies and little is known about its real incidence in this population of patients. Of note P3 muscle biopsy showed mitochondrial abnormalities consisting of cox-negative fibers and few mitochondrial aggregates that could be partly related with the age. Although histological findings may be very mild and even absent in genetically confirmed mitochondrial diseases, the clinical features presented by P3 oriented the genetic analysis toward mitochondrial diseases. Furthermore, P3 presented a long history of ischemic cardiopathy. *DNA2* expression in the heart is high, suggesting that mutations in *DNA2* can also affect the cardiac muscle. Recent evidences highlighted the key role of mitochondria in the pathogenesis of atherosclerosis and ischemic heart disease (1). Mitochondria disfunction lead to the accumulation of reactive oxygen species (ROS) in endothelial and smooth muscle cells, triggering vascular damage (16). Additionally, ROS can directly damage myocytes and contribute to impair cardiac function. DNA2 belongs to the helicase/nuclease family of proteins and its role is crucial in maintaining mtDNA integrity by repairing oxidative mtDNA damage. We can postulate that impaired DNA2 protein function increases oxidative mtDNA damage levels in cardiac tissue, driving to cardiac disfunction. Similarly, a polymorphic *WRN* variant, a member of the RecQ family of helicases implicated in maintaining genome stability, has been associated with increased risk of ischemic cardiopathy (9). Long PCR analysis on muscle samples from P3 revealed multiple mtDNA deletions, underlining the critical role of DNA2 in repairing mtDNA damages induced by oxidation. Eventually P3 was under treatment with statin when he experienced rhabdomyolysis. Statins are worldwide used to treat hypercholesterolemia. If the treatment is effective and safe for the majority of patients, a small percentage of them experience statin-induced myopathy, a condition characterized by various grade of muscle weakness, myalgia, and rhabdomyolysis (17). Reports from literature associate statin-induced myopathy with mitochondrial dysfunction and mitochondrial DNA (mtDNA) depletion (18) but not multiple deletions as presented by our patient. Statin use might unmask a mitochondrial subclinical dysfunction and we suppose that this individual susceptibility in P3 underlies the mechanism of inaugural rhabdomyolysis. We can also speculate that the pleiotropic effect of these compound exerted via protein hypoprenilation, could have contributed to unmask the DNA2 defect leading to rhabdomyolysis. A similar mechanism has been previously described in other metabolic myopathies (19). Clinicians should be precautious when treating with statins patients suspected of primary skeletal disease.

## Conclusions

With our work we enlarge the phenotypic and genetic spectrum of *DNA2* mutation.

## Data Availability Statement

The raw data supporting the conclusions of this manuscript will be made available by the authors, without undue reservation, to any qualified researcher.

## Ethics Statement

This study was approved and performed under the ethical guidelines issued by our institutions for clinical studies and was in compliance with the Helsinki Declaration. Informed written consent was obtained from all patients. Written informed consent to participate in this study was provided by the participants' legal guardian/next of kin. Written informed consent was obtained from the individual(s), and minor(s)' legal guardian/next of kin, for the publication of any potentially identifiable images or data included in this article.

## Author's Note

In ***Novel phenotypes associated to DNA2 mitochondrial disorders*** we describe three patients showing two novel mutations in *DNA2*. DNA replication helicase/nuclease 2 (DNA2) encodes for a helicase/nuclease family member involved in the processes of mtDNA replication and repair. *DNA2* gene associated mitochondrial myopathy was firstly described in four patients by Ronchi et al. as an adult onset skeletal myopathy involving the limb-girdle and lower limbs. Cardiomyopathy is a frequent finding in mitochondrial disease. To the present day, no reports are found about its association with *DNA2* mutations. With our paper, we alert the clinician about the possible cardiac involvement associated to *DNA2* related mitochondriopathy. We describe a novel phenotype of an early onset myopathy presenting with a velopharyngeal weakness as an associated feature and a late onset milder phenotype presenting with rhabdomyolysis. We think that the description of our cases might be helpful in diagnosing unsolved cases of early onset myopathy and we propose to consider rhabdomyolysis as a possible presentation mode for mitochondriopathies. We enlarge the genetic spectrum of *DNA2* mutations two novel mutations.

## Author Contributions

AG recruited and analyzed patients P1 and P2 wrote the manuscript. MB performed analyzed the data and contributed to the writing of the manuscript. SV-C, FF-V, and EK performed the histopathologic analysis of patients P1 and P2. RE analyzed patients. NR contributed to the histopathologic analysis of patient 3. CJ and BR performed the genetic analyses. TS analyzed one patient. EM conceived the study and design, supervised the study, performed and supervised the histopathology part of the study of patients 1 and 2, analyzed the data, and contributed to the writing of the manuscript. EM, AG, and MB conceived the manuscript, coordinated the collection and elaboration of data, and drafted the manuscript. EM and NR reviewed the muscles biopsies. SV-C, FF-V, EK, RE, AG, EM, TS, and MB provided patients data. AG, EM, BR, and CJ reviewed the genetic results. All the authors read and approved the manuscript.

### Conflict of Interest

The authors declare that the research was conducted in the absence of any commercial or financial relationships that could be construed as a potential conflict of interest.

## References

[1] Ahmed N Ronchi D Comi GP. Genes and pathways involved in adult onset disorders featuring muscle mitochondrial DNA instability. Int J Mol Sci. (2015) 16:18054–76. 10.3390/ijms160818054 26251896 PMC4581235

[2] Zheng L Zhou M Guo Z Lu H Qian L Dai H. Human DNA2 is a mitochondrial nuclease/helicase for efficient processing of DNA replication and repair intermediates. Mol Cell. (2008) 32:325–36. 10.1016/j.molcel.2008.09.024 18995831 PMC2636562

[3] Ding L Liu Y. Borrowing nuclear DNA helicases to protect mitochondrial DNA. Int J Mol Sci. (2015) 16:10870–7. 10.3390/ijms160510870 25984607 PMC4463680

[4] Duxin JP Dao B Martinsson P. Human Dna2 is a nuclear and mitochondrial DNA maintenance protein. Mol Cell Biol. (2009) 29:4274–82. 10.1128/MCB.01834-08 19487465 PMC2715806

[5] Phowthongkum P Sun A Novel truncating variant in DNA2-related congenital onset myopathy and ptosis suggest genotype-phenotype correlation. Neuromuscul Disord. (2017) 27:616–8. 10.1016/j.nmd.2017.03.013 28554558

[6] Khetarpal P Das S Panigrahi I Munshi A. Primordial dwarfism: overview of clinical and genetic aspects. Mol Genet Genomics. (2016) 291:1–15 10.1007/s00438-015-1110-y 26323792

[7] Ronchi D Di Fonzo A Lin W Bordoni A Liu C Fassone E. Mutations in DNA2 link progressive myopathy to mitochondrial DNA instability. Am J Hum Genet. (2013) 92:293–300. 10.1016/j.ajhg.2012.12.014 23352259 PMC3567272

[8] Chae JH Vasta V Cho A Lim BC Zhang Q Eun SH. Utility of a next generation sequencing in genetic diagnosis of early onset neuromuscular disorders. J Med Genet. (2014) 52:208–16. 10.1136/jmedgenet-2014-102819 25635128

[9] Ye L Miki T Nakura J Oshima J Kamino K Rakugi H. Association of a polymorphic variant of the Werner helicase gene with myocardial infarction in a Japanese population. Am J Med Genet. (1997) 68:494–8. 10.1002/(sici)1096-8628(19970211)68:4<494::aid-ajmg30>3.0.co;2-l 9021029

[10] Budd ME Campbell JL. Interplay of Mre11 nuclease with Dna2 plus Sgs1 in Rad51-dependent recombinational repair. PLoS ONE. (2009) 4:e4267. 10.1371/journal.pone.0004267 19165339 PMC2625443

[11] Andalib S Divani AA Michel TM Høilund-Carlsen PF Vafaee MS Gjedde A. Pandora's Box: mitochondrial defects in ischaemic heart disease and stroke. Expert Rev Mol Med. (2017) 19:e5. 10.1017/erm.2017.5 28376937

[12] Stöllberger C Finsterer J. Understanding left ventricular hypertrabeculation/noncompaction: pathomorphologic findings and prognostic impact of neuromuscular comorbidities. Expert Rev Cardiovasc Ther. (2019) 17:95–109. 10.1080/14779072.2019.1561280 30570401

[13] Hirano M Davidson M DiMauro S. Mitochondria and the heart. Curr Opin Cardiol. (2001) 16:201–10. 10.1097/00001573-200105000-00008 11357017

[14] Fratter C Gorman GS Stewart JD Buddles M Smith C Evans J. The clinical, histochemical, and molecular spectrum of PEO1 (Twinkle)-linked adPEO. Neurology. (2010) 74:1619–26. 10.1212/WNL.0b013e3181df099f 20479361 PMC2875130

[15] Spiegel R Saada A Flannery PJ Burté F Soiferman D Khayat M. Fatal infantile mitochondrial encephalomyopathy, hypertrophic cardiomyopathy and optic atrophy associated with a homozygous OPA1 mutation. J Med Genet. (2016) 53:127–31. 10.1136/jmedgenet-2015-103361 26561570 PMC4752660

[16] Buja LM. The pathobiology of acute coronary syndromes. Clinical implications and central role of the mitochondria. Tex Heart Inst J. (2013) 40:221–8. 23914009 PMC3709209

[17] Mohassel P Mammen AL. The spectrum of statin myopathy. Curr Opin Rhumatol. (2013) 25:747–52. 10.1097/01.bor.0000434673.85515.89 24061077

[18] Stringer HAJ Sohi GK Maguire JA Côté HCF. Decreased skeletal muscle mitochondrial DNA in patients with statin-induced myopathy. J Neurol Sci. (2013) 325:142–7. 10.1016/j.jns.2012.12.023 23312852

[19] Baker SK Vladutiu GD Peltier WL Isackson PJ Tarnopolsky MA. Metabolic myopathies discovered during investigations of statin myopathy. Can J Neurol Sci. (2008) 35:94–7. 10.1017/S0317167100007630 18380285

