# Supplementary material for: Novel Phenotypes and Cardiac Involvement Associated With DNA2 Genetic Variants
Source: Front Neurol. 2019 Oct 4;10:1049. doi: 10.3389/fneur.2019.01049 (PMC6787284; doi:10.3389/fneur.2019.01049)
Supplement: Supplementary file 1 [file Table_1.pdf]

Appendix 1

Table 1 is a list of all the 244 genes composing the next generation sequencing panel (NGS) that we used to perform molecular studies in P1, P2 and P3. The NGS panel (Roche NibelGen, Madison, WI, USA) comprises the entire coding region and exon–intron junctions of 244 genes associated with mitochondrial diseases. More than 95% of the nucleotides within the exons in the targeted regions are interrogated at more than 30× depth of coverage.

AARS2	NM_020745
AARS2	NM_020745
ABCB7	NM_004299.5
ACAD9	NM_014049
ACADM	NM_000016.5
ACADS	NM_000017.3
ACADSB	NM_001609.3
ACADVL	NM_000018.3
ACAT1	NM_000019.3
ACO2	NM_001098.2
ADCK3	NM_020247
ADCK4	NM_024876
AFG3L2	NM_006796
AGK	NM_018238
AIFM1	NM_001130846
AMACR	NM_014324.5
APTX	NM_001195248.1
ATP5A1	NM_001001937
ATP5E	NM_006886
ATPAF2	NM_145691
AUH	NM_001698.2
BCS1L	NM_001257342
BOLA3	NM_212552
BTD	NM_000060.2
C10ORF2 =TWNK	NM_021830
C12ORF65	NM_001143905.2
C20orf72=MGME1	NM_052865
CARS2	NM_024537
CEP89	NM_032816.4
CHCHD10	NM_213720
CHCHD3	NM_017812.3
CHCHD4	NM_144636.2

CHCHD6	NM_032343.2
CHKB	NM_005198
ChrM	NC_012920.1
CISD2	NM_001008388.4
CLPP	NM_006012.2
COA5	NM_001008215
COA6	NM_001206641
COASY	NM_001042532.3
COQ2	NM_015697
COQ4	NM_016035
COQ6	NM_182476
COQ9	NM_020312
COX10	NM_001303
COX14	NM_001257133
COX15	NM_078470
COX20	NM_198076
COX4I2	NM_032609
COX6B1	NM_001863
COX7B	NM_001866.2
CPT1A	NM_001876.3
CPT2	NM_000098.2
CRAT	NM_000755.4
CYC1	NM_001916
DARS2	NM_018122
DDHD1	NM_001160148
DDHD2	NM_015214
DGUOK	NM_080916
DLAT	NM_001931.4
DLD	NM_000108.4
DNA2	NM_001080449
DNAJC19	NM_145261.3
DNM1L	NM_012062
EARS2	NM_001083614
ECHS1	NM_004092.3
ELAC2	NM_018127.6
ERCC6	NM_000124.3
ETFA	NM_000126.3
ETFB	NM_001014763.1
ETFDH	NM_004453.3
ETHE1	NM_014297.4
FARS2	NM_001318872.1
FASTKD2	NM_001136193.1
FBP1	NM_000507.3
FBXL4	NM_012160
FDX1L	NM_001031734.3

FH	NM_000143.3
FIS1	NM_016068.2
FOXRED1	NM_017547
GAMT	NM_000156.5
GARS	NM_002047.2
GATM	NM_001482.2
GDAP1	NM_018972.2
GFER	NM_005262.2
GFM1	NM_001308164.1
GLRX5	NM_016417.2
GTPBP3	NM_001195422.1
HADHA	NM_000182.4
HADHB	NM_001281513.1
HARS2	NM_012208
HIBCH	NM_014362.3
HLCS	NM_000411.7
HMGCL	NM_000191.2
HMGCS2	NM_005518.3
HSD17B10	NM_004493.2
HTRA2	NM_013247.4
IARS2	NM_018060
IBA57	NM_001010867
IDH2	NM_002168.2
IDH3B	NM_006899.4
IMMT	NM_006839.2
ISCA1	NM_030940
ISCA2	NM_194279
ISCU	NM_213595
KARS	NM_001130089.1
KIF5A	NM_004984.3
L2HGDH	NM_024884.2
LARS	NM_020117
LARS2	NM_015340
LIAS	NM_006859.2
LRPPRC	NM_133259
LYRM4	NM_001164840
LYRM7	NM_181705
MARS2	NM_138395
MDH2	NM_005918.3
MFN1	NM_033540.2
MFN2	NM_014874
MGME1 (c20orf72)	NM_001310338.1
MICU1	NM_006077
MINOS1	NM_001204083.1

MIPEP	NM_005932
MPC1	NM_016098.3
MPV17	NM_002437
MRPL3	NM_007208.3
MRPL44	NM_022915.3
MRPS16	NM_016065
MRPS22	NM_020191.2
MRPS7	NM_015971
MTFMT	NM_139242
MTO1	NM_001123226
MTPAP	NM_018109
NDUFA1	NM_004541
NDUFA10	NM_004544
NDUFA11	NM_001193375
NDUFA11	NM_175614
NDUFA12	NM_018838
NDUFA2	NM_002488
NDUFA9	NM_005002
NDUFAF1	NM_016013
NDUFAF2	NM_174889
NDUFAF3	NM_199069
NDUFAF4	NM_014165
NDUFAF5	NM_024120
NDUFAF6	NM_152416
NDUFB3	NM_002491
NDUFB9	NM_005005
NDUFS1	NM_001199984
NDUFS2	NM_004550
NDUFS3	NM_004551
NDUFS4	NM_002495
NDUFS6	NM_004553
NDUFS7	NM_024407
NDUFS8	NM_002496
NDUFV1	NM_007103
NDUFV2	NM_021074
NFS1	NM_021100
NFU1	NM_001002755.2
NUBPL	NM_025152
OGDH	NM_002541.3
OPA1	NM_130837
OPA3	NM_001017989.2
OPA3	NM_025136.3
OXCT1	NM_000436.3
PANK2	NM_153638.2
PARK2	NM_004562.2

PC	NM_000920.3
PCK2	NM_004563.3
PDHA1	NM_001173454.1
PDHB	NM_000925.3
PDHX	NM_003477
PDK3	NM_001142386.2
PDP1	NM_001161779.1
PDSS1	NM_014317
PDSS2	NM_020381
PET100	NM_001171155
PINK1	NM_032409.2
PNPLA2	NM_020376.3
PNPT1	NM_033109
POLG	NM_002693
POLG2	NM_007215
PUS1	NM_025215
QIL1	NM_205767
QRSL1	NM_018292
RARS2	NM_020320
RMND1	NM_017909
RNASEH1	NM_002936
RRM2B	NM_001172477
SACS	NM_014363.4
SARS2	NM_001145901
SCO1	NM_004589
SCO2	NM_005138
SDHA	NM_004168
SDHAF1	NM_001042631
SDHAF2	NM_017841
SDHB	NM_003000
SDHC	NM_003001
SDHD	NM_003002
SERAC1	NM_032861
SETX	NM_015046
SFXN4	NM_213649.1
SLC19A2	NM_006996.2
SLC19A3	NM_025243
SLC22A5	NM_001308122.1
SLC25A1	NM_001256534.1
SLC25A12	NM_003705.4
SLC25A19	NM_001126121
SLC25A20	NM_000387
SLC25A22	NM_001191060
SLC25A3	NM_005888
SLC25A4	NM_001151

SLC25A46	NM_138773
SLC33A1	NM_001190992.1
SLC6A8	NM_005629.3
SPG7	NM_003119
SRY	NM_003140
SUCLA2	NM_003850
SUCLG1	NM_003849
SUCLG2	NM_001177599.1
SURF1	NM_003172
SYNE1	NM_182961.2
TACO1	NM_016360
TARS2	NM_025150
TAZ	NM_000116
TIMM8A	NM_004085
TK2	NM_004614
TMEM126B	NM_018480
TMEM240	NM_001114748
TMEM70	NM_017866
TPK1	NM_022445.3
TRIT1	NM_017646
TRMU	NM_018006.4
TSFM	NM_001172696
TTC11	NM_016068
TTC19	NM_017775
TUFM	NM_003321
TYMP	NM_001257989
UQCC2	NM_032340
UQCC3	NM_001085372
UQCRB	NM_001254752
UQCRC2	NM_003366
UQCRQ	NM_014402
VARS2	NM_001167734
WARS2	NM_015836
WFS1	NM_006005
YARS2	NM_001040436
LTBP4	NM_001042544.1

Table 2 is a list of VUS or possible pathogenic variant for P1 and P3. All these genes are autosomal recessive and all variants are heterozygous.

	Gene	HGVSc	HGVSp	Freq
P1	ACADS	c.65G>T	p.Trp22Leu	htz
	TUFM	c.760G>A	p.Ala254Thr	htz
	PANK2	c.1413-3_1413-2delCA	---	htz
P3	SACS	c.3427C>A	p.Gln1143Lys	htz
	TMEM126B	c.440T>C	p.Ile147Thr	htz
